# mTOR/HDAC1 Crosstalk Mediated Suppression of ADH1A and ALDH2 Links Alcohol Metabolism to Hepatocellular Carcinoma Onset and Progression *in silico*

**DOI:** 10.3389/fonc.2019.01000

**Published:** 2019-10-04

**Authors:** Kashif Rafiq Zahid, Shun Yao, Abdur Rehman Raza Khan, Umar Raza, Deming Gou

**Affiliations:** ^1^Shenzhen Key Laboratory of Microbial Genetic Engineering, Vascular Disease Research Center, College of Life Sciences and Oceanography, Provincial Key Laboratory of Regional Immunity and Diseases, Carson International Cancer Center, Shenzhen University, Shenzhen, China; ^2^Key Laboratory of Optoelectronic Devices and Systems of Ministry of Education and Guangdong Province, College of Optoelectronic Engineering Shenzhen University, Shenzhen, China; ^3^Department of Neurosurgery and Pituitary Tumor Center, The First Affiliated Hospital, Sun Yat-sen University, Guangzhou, China; ^4^Military College of Signals, National University of Science and Technology, Rawalpindi, Pakistan; ^5^Department of Biological Sciences, National University of Medical Sciences, Rawalpindi, Pakistan

**Keywords:** hepatocellular carcinoma, tumor progression, metabolic imbalance, mTOR signaling, alcohol metabolism, ADH1A, ALDH2

## Abstract

Hepatocellular carcinoma (HCC) is ranked the third deadliest cancer worldwide whose molecular pathogenesis is not fully understood. Although deregulated metabolic pathways have been implicated in HCC onset and progression, the mechanisms triggering this metabolic imbalance are yet to be explored. Here, we identified a gene signature coding catabolic enzymes (Cat-GS) involved in key metabolic pathways like amino acid, lipid, carbohydrate, drug, and retinol metabolism as suppressed in HCC. A higher expression of deregulated Cat-GS is associated with good survival and less aggressive disease state in HCC patients. On the other hand, we identified mTOR signaling as a key determinant in HCC onset and progression, whose hyperactivation is found associated with poor survival and aggressive disease state in HCC patients. Next, out of Cat-GS, we established two key regulators of alcohol metabolism, alcohol dehydrogenase 1A (ADH1A) and aldehyde dehydrogenase 2 (ALDH2), as being transcriptionally suppressed by histone deacetylase 1 (HDAC1) at the downstream of mTORC1 signaling. Suppressed ADH1A and ALDH2 expression aligns well with HCC-specific molecular profile and can efficiently predict disease onset and progression, whereas higher ADH1A and ALDH2 expression is associated with good survival and less aggressive disease state in HCC patients. Overall, our *in silico* findings suggest that transcriptional suppression of alcohol metabolism regulators, ADH1A and ALDH2, at the downstream of mTOR signaling is, in part, responsible for triggering oncogenic transformation of hepatocytes resulting in disease onset and progression in HCC.

## Introduction

Despite the great success witnessed in diagnosis, therapeutic treatment, and prevention over the past two decades, cancer is still among the deadliest diseases worldwide with more than 1.7 million new cases diagnosed and 0.6 million estimated deaths in the United States alone in 2018 (1). Hepatocellular carcinoma (HCC) is the most common and lethal primary hepatic malignancy with average survival rates varying between 6 and 20 months (2). Rise in chronic hepatitis B and C infections in recent years has likely triggered the increase in incidence rate of HCC around the globe (3). Being ranked sixth in term of incidence rate among all the malignancies worldwide, HCC stands as third leading cause of cancer attributed mortality (4). Although recent advancements in the area of functional genomics have greatly improved our knowledge, the molecular pathogenesis of HCC is not fully understood (5). Liver is the metabolic hub of the body and the deregulation of multiple metabolic processes has been suggested as key determinant of HCC onset and progression (6–8), but the mechanisms which trigger this metabolic imbalance in HCC are not well-understood. That's why a better understanding of molecular mechanisms behind HCC onset and progression is essential for effective diagnosis and therapeutic treatment.

Tumorigenesis may start upon genetic alterations of key regulators and effectors, resulting in aberrant regulation of signaling pathways. Chronic viral infection and/or exposure to hepatotoxic agents has been reported to initiate one or multiple signaling pathways in hepatocytes leading to their oncogenic transformation (9). Major pathways involved in HCC pathogenesis include pathways regulated by hepatocyte growth factor, epidermal growth factor, insulin-like growth factor, fibroblast growth factor, and platelet derived growth factor; angiogenic pathways regulated by vascular endothelial growth factor; and pathways related to cell differentiation such as Hedgehog, Notch, and WNT signaling pathways (10–12). Despite the vast variety of receptors to receive the growth signal at cell surface, the major signaling mediators downstream of the most of receptor tyrosine kinases (RTKs) are (i) MAPK/ERK signaling cascade and (ii) phosphoinositide 3-kinase (PI3K)/Akt/Mechanistic target of rapamycin (mTOR) signaling pathway in HCC (13, 14). Although the interplay of signaling cascades is well-understood in cancer, in general, and in HCC, in particular, the way in which these signaling pathways interact with the metabolic imbalance in HCC is not well-established (8). That is why a better understanding of signal transduction and identification of distant molecular targets at the downstream of signaling pathways is necessary to devise novel therapeutic approaches to treat HCC.

In this study, we aimed to identify the metabolic pathways consistently deregulated in HCC and to explore the molecular mechanisms triggering this metabolic imbalance in HCC. We identified multiple metabolic pathways associated with amino acid, lipid, carbohydrate, drug, and retinol metabolism as deregulated in HCC tumors compared to normal adjacent tissues by analyzing gene expression data of HCC patients from The Cancer Genome Atlas (TCGA) database. In-depth analysis of profiling data from multiple online available HCC patient datasets and from TCGA database revealed that a set of catabolic enzymes (Cat-GS) associated with abovementioned metabolic processes gets consistently downregulated in HCC. Notably, a higher expression of these catabolic enzymes is associated with good survival and a less aggressive disease state in HCC patients. On the other hand, we identified mTOR signaling as key determinant in HCC onset and progression whose hyperactivation is found associated with poor survival and aggressive disease state in HCC patients. Next, out of Cat-GS, we established two key regulators of alcohol metabolism, alcohol dehydrogenase 1A (ADH1A) and aldehyde dehydrogenase 2 (ALDH2) as being transcriptionally suppressed by histone deacetylase 1 (HDAC1) at the downstream of mTORC1 signaling. A higher expression of ADH1A and ALDH2 is associated with good survival and a less aggressive disease state in HCC patients.

## Materials and Methods

### Data Retrieved

Gene expression data were retrieved from the National Center for Biotechnology Information (NCBI) Gene Expression Omnibus (GEO) database [GSE12941 (15), GSE15654 (16), GSE17856 (17), GSE20017 (18), GSE25097 (19), GSE29721 (20), GSE36376 (21), GSE37129 (22), GSE39791 (23), GSE43619 (24), GSE45436 (25), GSE47197, GSE54236 (26), GSE55092 (27), GSE57555 (28), GSE57957 (29), GSE59713 (30), GSE62232 (31), GSE64041, GSE76297 (32), GSE76427 (33), GSE77322 (34), GSE79246 (35), GSE84402 (36), GSE84598 (37), GSE85257 (35), and GSE87630 (38)]. Gene methylation data were retrieved from the NCBI GEO database [GSE37988 (39), GSE44909 (40), GSE57956 (29)]. In addition, gene methylation, gene expression and Reverse Phase Protein Array (RPPA) data of HCC patients at TCGA database were retrieved online from Broad GDAC Firehose website: https://gdac.broadinstitute.org/. Summary of patient's datasets analyzed for molecular and clinico-pathological comparisons is available in Table S1. ADH1A and ALDH2 gene mutation profile of HCC patients at TCGA database were retrieved online from cBioPortal for cancer genomics: https://www.cbioportal.org/. Immunohistochemistry (IHC) data for mTOR, ADH1A, and ALDH2 protein expression in HCC tissues was retrieved online from The Human Protein Atlas: https://www.proteinatlas.org/. Data for motif based binding of transcription and chromatin remodeling factors within 1 kb up- and downstream of transcription start site of ADH1A and ALDH2 was retrieved online from ChIPBase v2.0: http://rna.sysu.edu.cn/chipbase.

### Identification of Differentially Expressed Genes and KEGG Pathway Enrichment Analyses

Patients' gene expression data retrieved from NCBI GEO database (GSE76297, GSE76427, GSE84402, and GSE84598), were first normalized by calculating *Z*-scores for each gene within individual datasets. Later, these normalized data were pooled and gene expressions were compared between HCC and adjacent normal tissues using two tailed Student's *t*-test. Bonferroni adjustment was applied to exclude false positives. Significance cut-off was taken as Bonferroni adjusted *p* < 0.05. Lists of top significantly upregulated (*n* = 1,000) and downregulated (*n* = 1,000) genes were separately uploaded to online freely available DAVID functional annotation tool (https://david.ncifcrf.gov/summary.jsp) to identify Kyoto Encyclopedia of Genes and Genomes (KEGG) pathways suppressed or hyperactive in HCC tumors compared to adjacent normal tissues.

### Generating Catabolic and Other Gene Signatures

**Figure 1:**
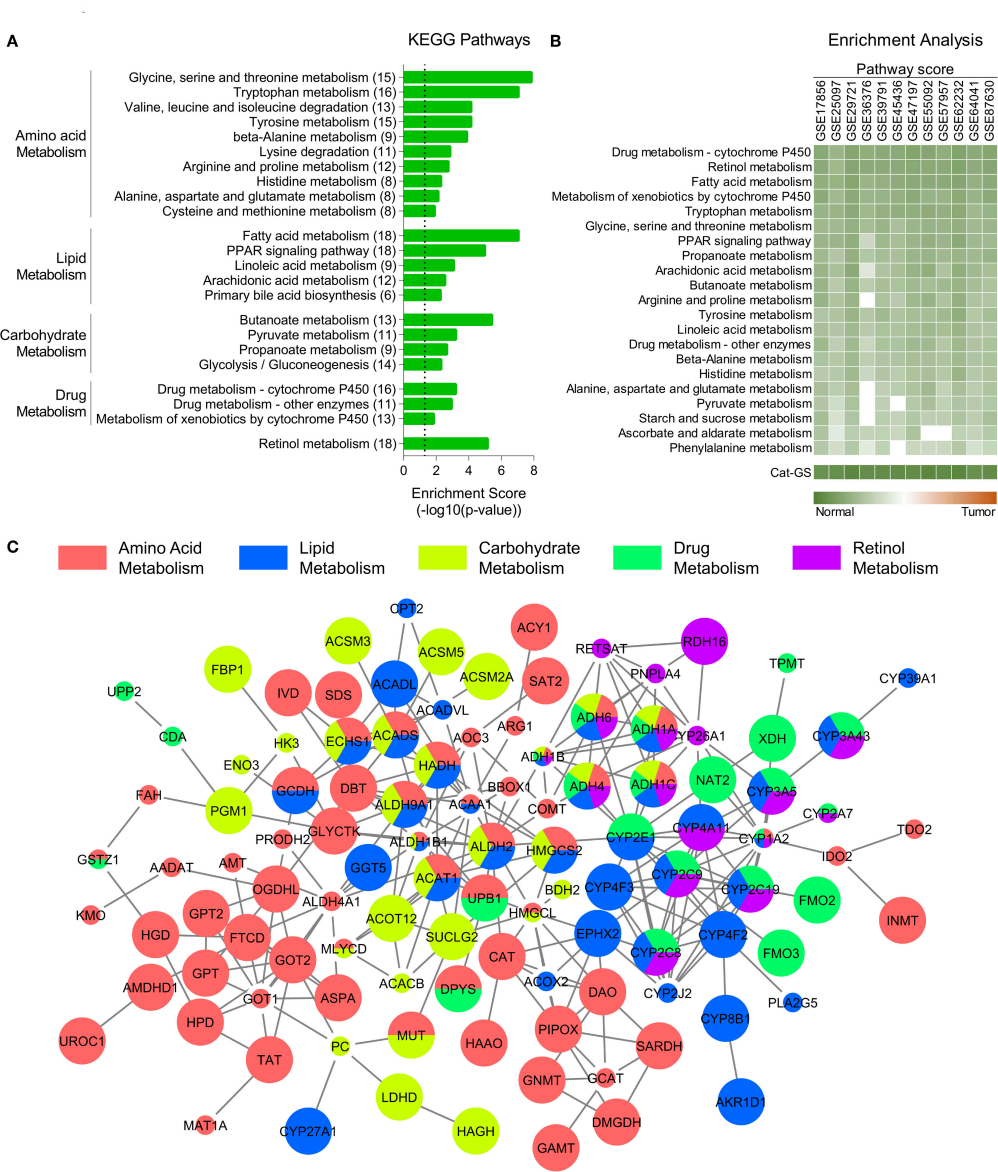
Catabolic enzymes associated with amino acid, lipid, carbohydrate, drug, and retinol metabolism are suppressed in HCC. **(A)** Bar-graph showing top KEGG pathways associated with genes downregulated in HCC tumors compared to adjacent normal tissues. **(B)** Heatmap showing enrichment of metabolic pathways associated gene sets and of catabolic gene signature (Cat-GS) in HCC or adjacent normal tissues of patients from 12 different GEO datasets. **(C)** Network showing interactions between the genes from Cat-GS. Node size, big and small, respectively, shows whether the gene is associated with survival in HCC patients or not. Node color shows the involvement of gene in one or more of five major metabolic processes downregulated in HCC.

Among the top significantly downregulated (*n* = 1,000) genes, 159 were identified as being involved in top deregulated metabolic pathways (Figure 1A) in HCC tumors compared to adjacent normal tissues. As metabolic pathways mainly work through reactions carried out by anabolic and catabolic enzymes, we looked for genes coding metabolic enzymes and shortlisted 119 genes as having enzymatic function. Next, the type of function (anabolic/catabolic) for each of these gene was identified through web and literature based search. Interestingly, more than 90% of these genes were coding catabolic enzymes (*n* = 110). These catabolic enzyme coding genes were combined to make a catabolic gene signature (Cat-GS). Five smaller gene signatures representing each of five major metabolic processes suppressed in HCC tumors compared to adjacent normal tissues were generated from Cat-GS by taking out the list of genes associated with each process. These gene signatures were named as amino acid (AA_Met-GS), lipid (Lipid_Met-GS), carbohydrate (Carb_Met-GS), drug (Drug_Met-GS), and retinol (Retinol_Met-GS) metabolism gene signatures. Alcohol metabolism associated gene signature (Alco_Met-GS) was generated by combining the two alcohol metabolism associated genes, ADH1A and ALDH2, found to be regulated at the downstream of mTOR signaling.

### Constructing Interactive Network for Cat-GS

List of genes in Cat-GS was uploaded to the Search Tool for the Retrieval of Interacting Genes (STRING) database (https://string-db.org). A high confidence threshold of 0.7 was used to identify the true protein-protein interactions between the genes from Cat-GS. Interaction file was then exported and a replica of network was created using Cytoscape software v3.7.1. In cytoscape, nodes were colored based on their involvement in any of five major metabolic processes suppressed in HCC tumors compared to adjacent normal tissues. In addition, genes whose expression was associated with survival in HCC patients from TCGA database were shown with bigger nodes as compared to those whose expression was not associated with survival.

### Patient Score, Pathway Score, and Enrichment Analyses

Gene sets related to metabolic processes, liver cancer onset and progression, signaling pathways, HDAC1 activity, and cell cycle were downloaded from the GSEA and KEGG portals. Z-scores were calculated for each gene within all patients' datasets. Patient score was calculated by summing the *Z*-scores for all the genes in a specific gene set for that patient (8, 41). HDAC1 activity score for each patient was calculated by taking average of scores of three different gene sets related to HDAC1 activity. In order to calculate pathway scores, patients were divided into two groups (G0 and G1) depending upon type of analyses i.e., “Normal (G0) vs. Tumor (G1),” “Low Cat-GS (G0) vs. High Cat-GS (G1),” “Low mTOR expression (G0) vs. High mTOR expression (G1),” and “Low Alco_Met-GS (G0) vs. High Alco_Met-GS (G1).” Later on, patient scores from each group were averaged and the absolute difference between groups was calculated. Pathway score was then calculated by taking log of absolute average difference.

$$\begin{array}{l} Pathway\;score={\text{log}}_{10}\;\left[Abs\left({G0}_{avg}-{G1}_{avg}\right)\right] \end{array}$$

For pathway enrichment, patient scores between groups were compared using Student's *t*-test with significance cutoff *p* < 0.05. In order to give dimension to the enrichment, the pathway score was multiplied by −1 if average patient score in G0 was higher than that in G1. On the other hand, pathway score was multiplied by 1 if average patient score in G0 was less than that in G1. If the pathway was not enriched in any group i.e., *p* > 0.05, the pathway score was multiplied by 0. Pathway scores for the gene signatures, we developed, were also calculated in the same way as mentioned above. Geneset enrichment analysis (GSEA) was performed using HCC patients' data combined for meta-analysis and data from TCGA database, GSE25097, and GSE36376.

### Survival Analysis

All the survival analyses for patients from GEO dataset GSE15654 and from TCGA database were performed using R script (42). Survival curves were generated using Kaplan-Meier method. Patients without any available survival time or event were excluded from the corresponding patient groups. Significance of the difference in survival between groups was calculated by Log-rank (Mantel-Cox) test with a *p*-value cutoff of *p* < 0.05.

### Statistical Analyses

Venn diagrams were prepared using the online freely available tool at http://bioinformatics.psb.ugent.be/webtools/Venn/. Pearson correlation coefficients were calculated for correlation analyses. Comparison between two groups were made using two tailed Student's *t*-test. Significance cut-off for correlation analyses and Student's *t*-test was taken as *p* < 0.05. A single expression value was available for the samples in GSE77322, −1.96 > *Z*_diff_ > 1.96 threshold was used to identify differentially expressed genes (43).

## Results

### Catabolic Enzymes Associated With Amino Acid, Lipid, Carbohydrate, Drug, and Retinol Metabolism Are Suppressed in HCC

To identify the molecular players of disease onset and progression in HCC, we performed a meta-analysis using gene expression data of HCC tumors and adjacent normal tissues (Figure S1; For details, see Materials and Methods section) and identified significantly downregulated genes in HCC tumors compared to adjacent normal tissues. KEGG pathway enrichment analyses of these genes revealed that multiple metabolic pathways associated with amino acid, lipid, carbohydrate, drug, and retinol metabolism are suppressed in HCC tumors compared to adjacent normal liver tissues (Figure 1A). Although metabolic pathways involve both anabolic and catabolic enzymes, we found that the genes, which are both downregulated in HCC and are associated with abovementioned pathways, mainly encode catabolic enzymes (110 out of 159) (For details, see Materials and Methods section) suggesting that catabolic functions are suppressed in HCC. That's why we developed a catabolic gene signature (Cat-GS) comprising of these 110 genes as a signature of HCC onset and progression. In order to validate our findings, we calculated pathway scores (For details, see Materials and Methods section) of different gene sets representing amino acid, lipid, carbohydrate, drug, and retinol metabolism for 12 different GEO datasets where profiling data of HCC tumors and of adjacent normal liver tissues is available, and performed enrichment analysis. All these gene sets are significantly enriched in normal liver tissues compared to HCC tumors, confirming that metabolic imbalance is a key feature of HCC onset and progression (Figure 1B). In addition, with highest pathways scores observed, Cat-GS is also significantly enriched in normal liver tissues compared to HCC in all the datasets (Figure 1B), suggesting that the gene signature (Cat-GS) we developed has greater sensitivity and specificity to identify HCC onset and progression compared to any other gene set representing individual metabolic pathways. Interestingly, all the genes in Cat-GS are interrelated with one or another (Figure 1C; For details, see Materials and Methods section) and higher expression of most of these genes (70 out of 110) is associated with good overall survival in HCC patients from TCGA database (Figure 1C and Table S2).

### Cat-GS Is Associated With Less Aggressive Disease State and Good Survival in HCC Patients

**Figure 2:**
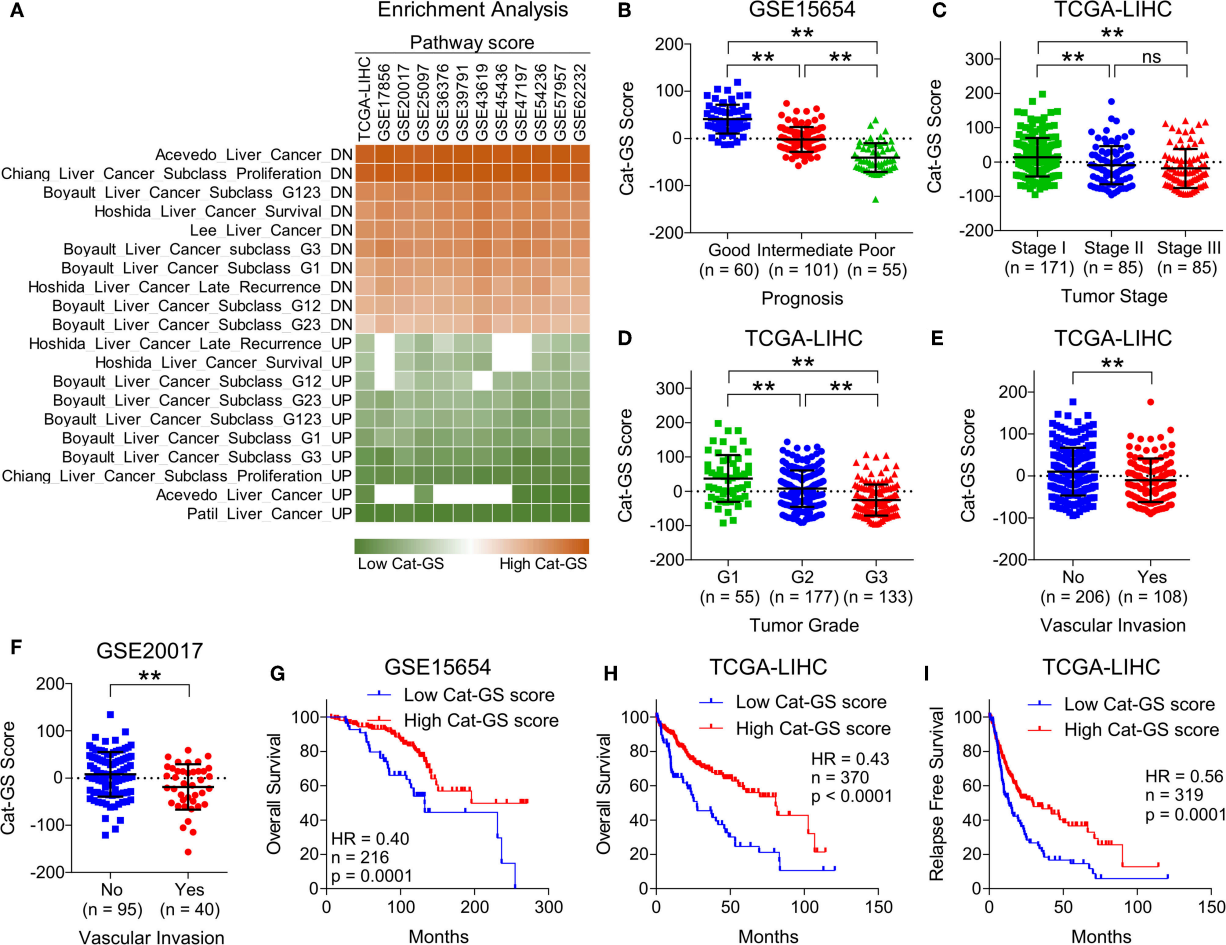
Cat-GS is associated with less aggressive state and good survival in HCC patients. **(A)** Heatmap showing enrichment of gene sets associated with liver cancer onset and progression in patients from TCGA database and from 11 different GEO datasets having low or high Cat-GS score. **(B)** Dot-plot showing Cat-GS scores in HCC patients from GEO dataset GSE15654 having good, intermediate or poor prognosis. **(C,D)** Dot-plots showing Cat-GS scores in HCC patients from TCGA database representing different tumor stages (stage I, II or III) **(C)** and different tumor grades (G1, G2, or G3) **(D)**. **(E,F)** Dot-plots showing Cat-GS scores in HCC patients from TCGA database **(E)** and from GEO dataset GSE20017 **(F)** who experienced or did not experience vascular invasion. **(G,H)** Kaplan-Meier survival plots representing the percentage overall survival in HCC patients from GEO dataset GSE15654 (*n* = 216) **(G)** and from TCGA database (*n* = 370) **(H)** based on low vs. high Cat-GS score. **(I)** Kaplan-Meier survival plot representing the percentage relapse free survival in HCC patients from TCGA database (*n* = 319) based on low vs. high Cat-GS score. **P < 0.01; ns, not significant, Student's *t*-test.

To validate the clinical significance of Cat-GS, we performed enrichment analysis in patients from TCGA database and from 11 different GEO datasets, and found that the genes which are downregulated during liver cancer onset and progression are significantly enriched in patients having high Cat-GS score whereas the genes which are upregulated during liver cancer onset and progression are significantly enriched in patients having low Cat-GS score (Figure 2A). Next, we found that Cat-GS is negatively correlated with prognosis in HCC as Cat-GS score is low in patients, having intermediate and poor prognosis compared to those having good prognosis (Figure 2B). We also found low Cat-GS score in Stage III and Grade 3 tumors compared to Stage I and Grade 1 tumors, respectively (Figures 2C,D). In two separate patient datasets, we observed significantly low Cat-GS score in patients who experienced vascular invasion compared to those having no signs of vascular invasion (Figures 2E,F). Notably, we observed that a high Cat-GS score is associated with good overall survival and relapse free survival in HCC patients from GEO dataset GSE15654 and TCGA database (Figures 2G–I). As Cat-GS comprised of overlapping and non-overlapping genes involved in amino acid, lipid, carbohydrate, drug, and retinol metabolism, we developed five sub gene signatures from Cat-GS representing each of these metabolic processes (For details; please see Materials and Methods section) and tested their association with disease state and survival in HCC patients. Interestingly, all these gene signatures were individually associated with less aggressive disease state and good survival in HCC patients as well (Figure S2). These results confirm that the suppression of metabolic pathways involving amino acid, lipid, carbohydrate, drug, and retinol metabolism is associated with an aggressive disease state in HCC and restoring these metabolic pathways would lead to good survival in HCC patients.

### mTOR Signaling Is Hyperactive and Is Associated With Aggressive Disease State and Poor Survival in HCC Patients

**Figure 3:**
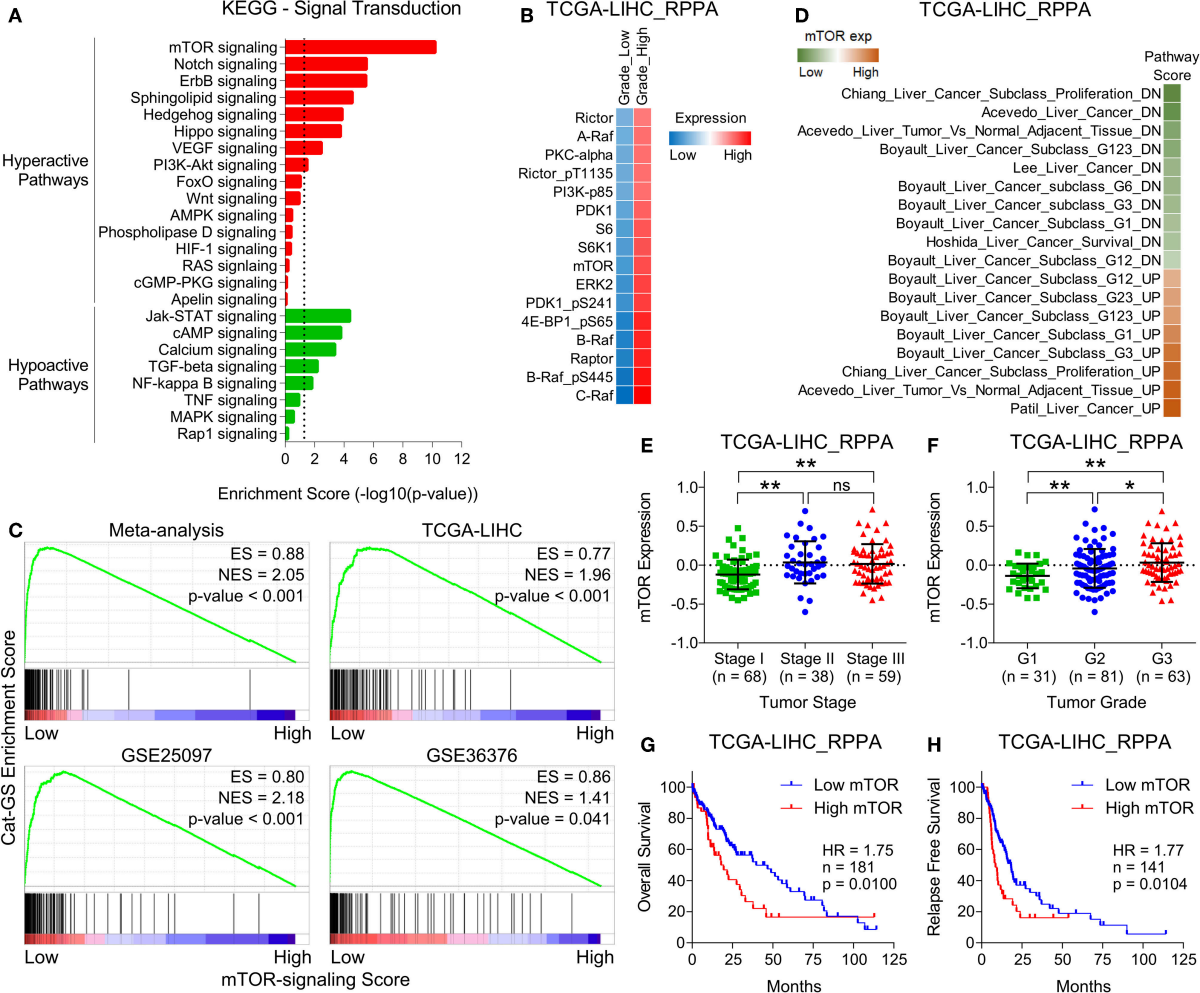
mTOR signaling is hyperactive and is associated with aggressive disease state and poor survival in HCC patients. **(A)** Bar-graph showing enrichment of signal transduction pathways in HCC tumors compared to adjacent normal tissues. **(B)** GSEA plots showing Cat-GS enrichment in HCC patients having low or high mTOR-signaling score. Plots were created using HCC patients data combined for meta-analysis (top left), and data from TCGA database (top right), GSE25097 (bottom left), and GSE36376 (bottom right). **(C)** Heatmap showing changes in protein expression of different upstream regulators and downstream effectors of mTOR signaling between low and high grade tumors of HCC patients from TCGA database. **(D)** Heatmap showing enrichment of gene sets associated with liver cancer onset and progression in patients from TCGA database having low or high mTOR protein expression. **(E,F)** Dot-plots showing mTOR protein expression in HCC patients from TCGA database representing different tumor stages (stage I, II or III) **(E)** and different tumor grades (G1, G2, or G3) **(F)**. **(G,H)** Kaplan-Meier survival plots representing the percentage overall survival (*n* = 181) **(G)** and percentage relapse free survival (*n* = 141) **(H)** in HCC patients from TCGA database based on low vs. high mTOR protein expression. *P < 0.05; **P < 0.01; ns, not significant, Student's *t*-test.

Next, we aimed to identify how genes involved in the metabolic processes get downregulated in HCC and hypothesized that the signaling pathways upregulated in HCC tumors compared to normal liver tissues might negatively regulate metabolic processes. In this line, we obtained lists of genes associated with different signaling pathways from KEGG signal transduction module and performed pathway enrichment analysis (Figure 3A) using same combined gene expression data we previously used to identify significantly downregulated genes in HCC (Figure 1). This analysis revealed mTOR signaling as top hyperactive pathway in HCC tumors compared to adjacent normal tissues (Figure 3A). Growth signals received by RTKs at the cell surface lead to activation of mTOR signaling at the downstream of PI3K/Akt pathway (13). Notably, PI3K/Akt signaling is also among the pathways hyperactive in HCC tumors compared to adjacent normal tissues (Figure 3A). mTOR is an evolutionary conserved serine/threonine protein kinase involved in regulation of broad spectrum of cellular processes including cell growth, aging, and metabolism (44). mTOR mainly works through making two distinct multiprotein complexes, namely mTORC1 and mTORC2 (45). The former one is mainly regulated via inhibition by tuberous sclerosis complex 1 (TSC1) and TSC2. MAPK/ERK pathway also converges to mTOR signaling via actively inhibiting TSC1/2. As a result, uninhibited mTORC1 promotes energy metabolism, protein synthesis, lipogenesis, cellular growth, and proliferation through its immediate downstream effectors including P70 S6 kinase (S6K1), 4E-binding protein-1 (4EBP1), and cyclin dependent kinases (46). Compared to mTORC1, less is known about how mTORC2 is activated but Akt has been established as directly activated at the downstream of mTORC2 (47) suggesting a complex interplay within PI3K/Akt/mTOR signaling pathway. Interestingly, the protein expression of multiple upstream regulators and downstream effectors of mTOR signaling is significantly upregulated in HCC tumors of high grade compared to those of low grade in patients from TCGA database (Figure 3B). In order to investigate whether upregulated mTOR signaling is involved in suppressing metabolic processes and inhibiting Cat-GS expression, we performed Gene Set Enrichment Analyses (GSEA) and found that Cat-GS is enriched in patients having low mTOR signaling score as compared to those having high mTOR signaling score from four different HCC patients datasets (Figure 3C) suggesting that mTOR-signaling might regulate Cat-GS during HCC onset and progression. Before moving forward to find that how this happens, we aimed to validate the clinical significance of mTOR signaling in HCC. In order to do this, we performed enrichment analysis using gene sets associated with liver cancer onset and progression, and found that the genes which are downregulated during liver cancer onset and progression are significantly enriched in patients having low mTOR protein expression, whereas, the genes which are upregulated during liver cancer onset and progression are significantly enriched in patients having high mTOR protein expression (Figure 3D). We also observed high mTOR protein expression and mTOR signaling score in Stage III and Grade 3 tumors compared to Stage I and Grade 1 tumors, respectively (Figures 3E,F and Figures S3A,B). In addition, we observed significantly high mTOR signaling score in patients who experienced vascular invasion compared to those having no signs of vascular invasion (Figure S3C). Notably, we found that high protein expression of mTOR, and multiple upstream regulators and downstream effectors of mTOR signaling is associated with poor overall survival and relapse free survival in HCC patients from TCGA database (Figures 3G,H and Figure S3D). In addition, high mTOR signaling score is also associated with poor overall survival and relapse free survival in HCC patients from TCGA database (Figures S3E,F). Overall, these results confirm that the hyperactivation of mTOR signaling is a key determinant in HCC onset and progression.

### HDAC1 Inhibits Alcohol Metabolism by Transcriptionally Suppressing ADH1A and ALDH2 at the Downstream of mTOR Signaling in HCC

**Figure 4:**
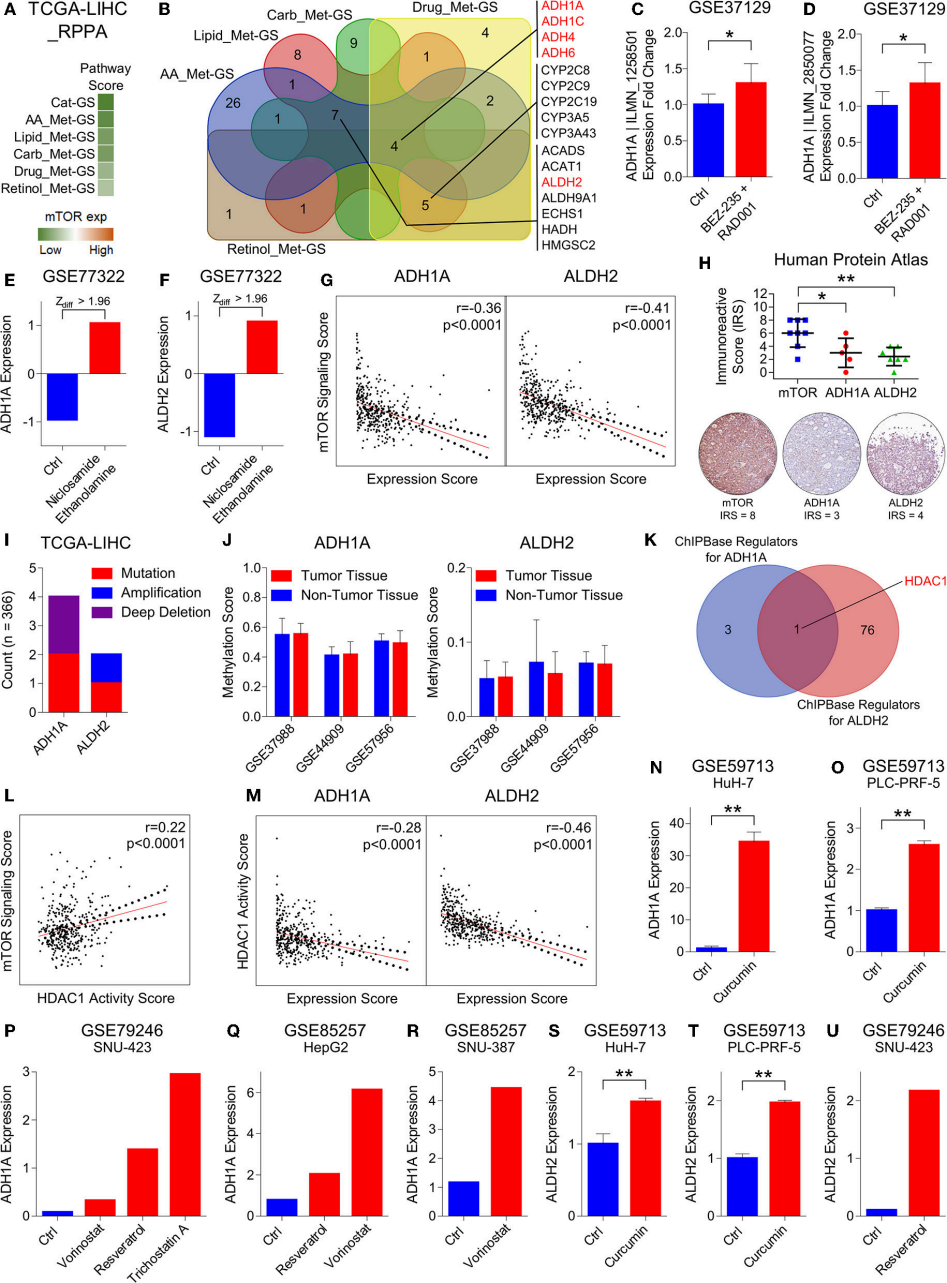
HDAC1 inhibits alcohol metabolism by transcriptionally suppressing ADH1A and ALDH2 at the downstream of mTOR signaling in HCC. **(A)** Heatmap showing enrichment of Cat-GS and gene signatures representing amino acid (AA_Met-GS), lipid (Lipid_Met-GS), carbohydrate (Carb_Met-GS), drug (Drug_Met-GS), and retinol (Retinol_Met-GS) metabolism in patients from TCGA database having low or high mTOR protein expression. **(B)** Venn diagram showing number of shared catabolic enzymes (which are also associated with survival in HCC patients) between gene signatures representing amino acid (AA_Met-GS), lipid (Lipid_Met-GS), carbohydrate (Carb_Met-GS), drug (Drug_Met-GS), and retinol (Retinol_Met-GS) metabolism. **(C,D)** Bar-graph showing changes in ADH1A | ILMN_1258501 **(C)** and ADH1A | ILMN_2850077 **(D)** expression in mouse model of HCC upon combinatorial treatment with BEZ-235 and RAD001 in GEO dataset GSE37129. **(E,F)** Bar-graph showing changes in ADH1A **(E)** and ALDH2 **(F)** expression in HepG2 cells upon treatment with Niclosamide Ethanolamine in GEO dataset GSE77322. **(G)** Graphs showing correlation of mTOR signaling sore with ADH1A (left) and ALDH2 (Right) expression in patients from TCGA database. **(H)** Dot-plot showing immunoreactive score of mTOR, ADH1A, and ALDH2 in HCC tissues of patients from Human Protein Atlas database (top). Representative IHC images of mTOR, ADH1A and ALDH2 protein expression in patient # 3324 from Human Protein Atlas database (below). **(I)** Bar-graph showing number of patients from TCGA database having mutation, amplification or deep deletion in ADH1A and ALDH2 genes. **(J)** Bar-graphs showing changes in methylation pattern of ADH1A (left) and ALDH2 (right) between HCC tumors and adjacent normal liver tissues in patients from GEO datasets GSE37988, GSE44909, and GSE57956. **(K)** Venn diagram showing number of shared transcriptional and chromatin regulators which can bind within 1 kb up- or downstream of transcription start site of ADH1A and ALDH2. **(L)** Graph showing correlation of mTOR signaling score with HDAC1 activity score in patients from TCGA database. **(M)** Graphs showing correlation of HDAC1 activity sore with ADH1A (left) and ALDH2 (Right) expression in patients from TCGA database. **(N,O)** Bar-graphs showing changes in ADH1A expression in HuH-7 **(N)** and PLC-PRF-5 **(O)** cells upon treatment with curcumin in GEO dataset GSE59713. **(P)** Bar-graph showing changes in ADH1A expression in SNU-423 cells upon treatment with vorinostat, resveratrol and trichostatin A in GEO dataset GSE79246. **(Q)** Bar-graph showing changes in ADH1A expression in HepG2 cell upon treatment with Resveratrol and vorinostat in GEO dataset GSE85257. **(R)** Bar-graph showing changes in ADH1A expression in SNU-387 cell upon treatment with vorinostat in GEO dataset GSE85257. **(S,T)** Bar-graphs showing changes in ALDH2 expression in HuH-7 **(S)** and PLC-PRF-5 **(T)** cells upon treatment with curcumin in GEO dataset GSE59713. **(U)** Bar-graph showing changes in ALDH2 expression in SNU-423 cells upon treatment with resveratrol in GEO dataset GSE79246. *P < 0.05; **P < 0.01; ns, not significant, Student's *t*-test.

The first line of evidence in the context that mTOR signaling might regulate metabolic processes downregulated in HCC came from GSEAs where we found that Cat-GS is enriched in patients having low mTOR signaling score as compared to those having high mTOR signaling score (Figure 3C). In order to further validate, we performed enrichment analysis using gene sets representing amino acid, lipid, carbohydrate, drug, and retinol metabolism, and found that all these gene sets are significantly enriched in patients having low mTOR expression compared to those having high mTOR expression (Figure S4). In addition, Cat-GS and other metabolic gene signatures we developed are also significantly enriched in patients having low mTOR expression compared to those having high mTOR expression confirming that metabolic processes understudy get suppressed at downstream of mTOR signaling (Figure 4A). In order to identify the functional metabolic targets of mTOR signaling, we first shortlisted the genes associated with survival in HCC patients from Cat-GS and then looked for the genes which are involved in multiple metabolic processes downregulated in HCC. As a result, 16 hits came as involved in 3 or more metabolic processes (Figure 4B). Interestingly, this list is enriched with genes involved in alcohol metabolism. In liver cells, alcohol is metabolized in a two-step process. During the first step, ethanol is metabolized to acetaldehyde by alcohol dehydrogenases, whereas in second step, acetaldehyde is metabolized to acetate by aldehyde dehydrogenases (48).

Next, we aimed to check which of these alcohol metabolism associated genes are regulated by mTOR signaling. In this line, we found that ADH1A expression gets upregulated in mouse model of HCC upon combinatorial treatment with PI3K inhibitor, BEZ-235, and mTOR inhibitor, RAD001 in GEO dataset GSE37129 (Figures 4C,D). Recently, niclosamide and its ethanolamine salt have been shown as anticancer agents and therapy sensitizers by inhibiting mTORC1 in multiple cancer types (49–51). By analyzing GEO dataset GSE77322, we found that the expression of two alcohol metabolism associated genes, ADH1A and ALDH2, gets significantly upregulated in HepG2 cells upon treatment with niclosamide ethanolamine (Figures 4E,F). We also found an inverse correlation of mTOR signaling score with ADH1A and ALDH2 expression in patients from TCGA database (Figure 4G) and from different GEO datasets (Figure S5A). In addition, IHC analysis of HCC tissues also revealed that high mTOR protein expression is associated with low protein expression of ADH1A and ALDH2 (Figure 4H). All these results suggested ADH1A and ALDH2 as being regulated at the downstream of mTOR signaling.

In order to rule out that changes in the expression pattern of ADH1A and ALDH2 during HCC onset and progression is due to genetic mutations, we checked their mutation level in HCC patients and found that these genes are mutated in <2% of HCC patients from TCGA database (Figure 4I). In addition to genetic mutations, changes in DNA methylation pattern also plays an important role in transcriptional regulation of gene expression. Interestingly, mTOR signaling has been reported to induce hypermethylation of different tumor suppressor genes including PTEN via upregulating DNA methyltransferases (52, 53). In this line, we checked the changes in DNA methylation pattern of ADH1A and ALDH2 genes between HCC tumor and adjacent normal liver tissues in patients from GEO datasets GSE37988, GSE44909, and GSE57956 but found no significant differences (Figure 4J) suggesting that ADH1A and ALDH2 expression is not regulated through DNA methylation during HCC onset and progression.

Lastly, in the quest to find DNA binding proteins including transcription factors and chromatic remodeling factors which can bind to or nearby transcription start sites of ADH1A and ALDH2 and regulate their expression, we found that HDAC1 is a common regulator of ADH1A and ALDH2 expression (Figure 4K). Interestingly, increased HDAC1 activity due to its phosphorylation by S6K1 at the downstream of mTORC1 signaling pathway has been shown to inhibit estrogen receptor-alpha expression; thus leading to therapy resistance in breast cancer (54). We also found a positive correlation between mTOR signaling score and HDAC1 activity score (For Details, see Materials and Methods section) in HCC patients from TCGA database (Figure 4L) and from different GEO datasets (Figure S5A). In addition, S6K1 protein expression is also positively correlated with HDAC1 activity in HCC patients from TCGA database (Figure S5B). Similar to mTOR expression and signaling, HDAC1 activity score was also associated with aggressive disease state and poor survival in HCC patients (Figures S5C–G) confirming its role in HCC onset and progression. In addition, HDAC1 activity score was inversely correlated with ADH1A and ALDH2 expression in HCC patients from TCGA database (Figure 4M) and from different GEO datasets (Figure S5H). These results were further confirmed by analyzing multiple GEO datasets where we found that ADH1A and ALDH2 expression gets upregulated in different HCC cell lines upon treatment with different drugs [Curcumin (55), Vorinostat (56), Resveratrol (57) and Trichostatin A (58)] known to inhibit HDAC1 or its activity. All these results confirm that two alcohol metabolism associated genes, ADH1A and ALDH2, are transcriptionally suppressed by HDAC1 at the downstream of mTORC1 signaling in HCC.

### Suppressed Alco_Met-GS Aligns Well With HCC-specific Molecular Profile

**Figure 5:**
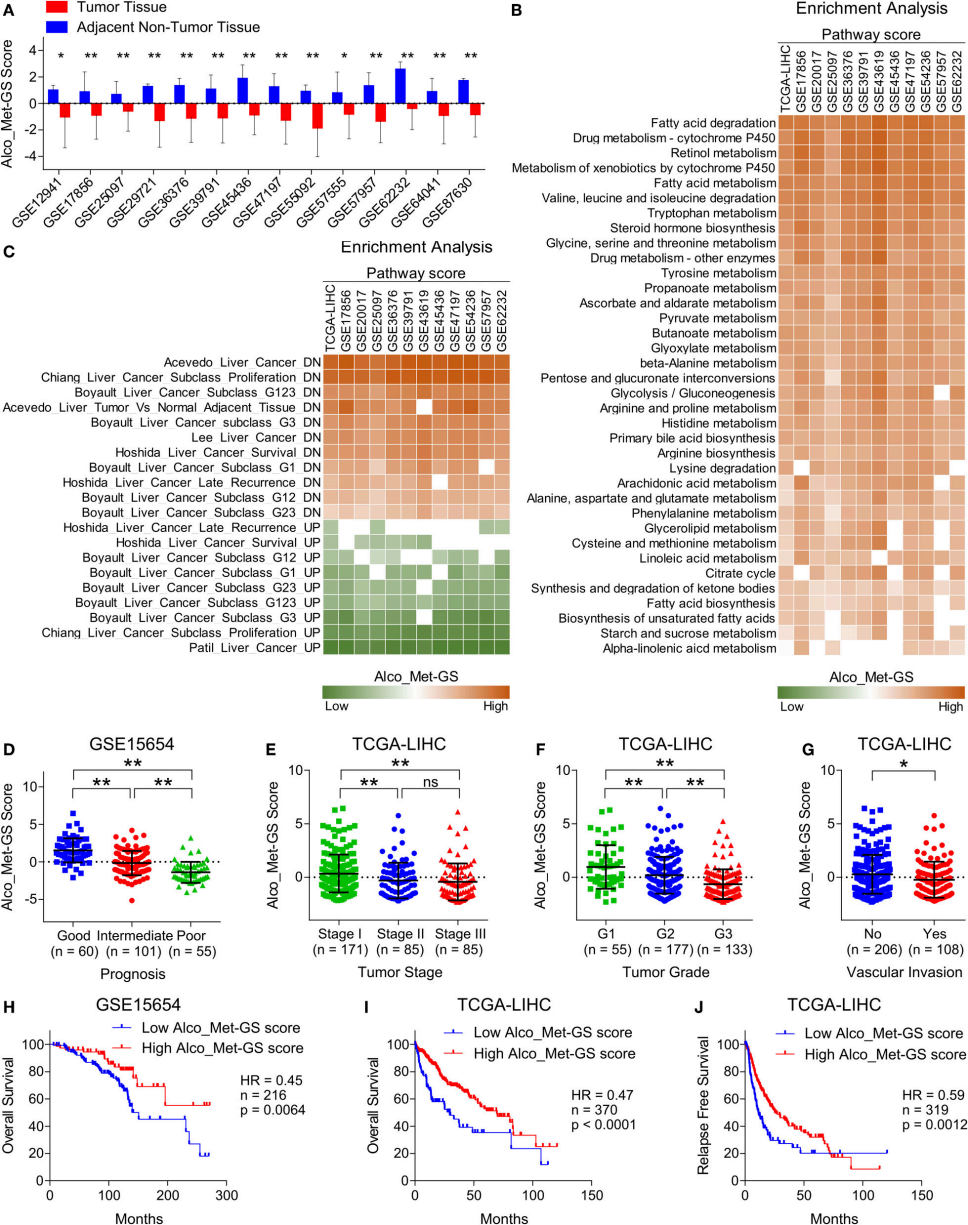
Suppressed Alco_Met-GS aligns well with HCC-specific molecular profile and its higher expression is associated with less aggressive disease state and good survival in HCC patients. **(A)** Bar-graph showing changes in Alco_Met-GS score between HCC tumors and adjacent normal tissues in patients from 14 different GEO datasets. **(B)** Heatmap showing enrichment of gene sets associated with metabolic pathways in patients from TCGA database and from 11 different GEO datasets having low or high Alco_Met-GS score. **(C)** Heatmap showing enrichment of gene sets associated with liver cancer onset and progression in patients from TCGA database and from 11 different GEO datasets having low or high Alco_Met-GS score. **(D)** Dot-plot showing Alco_Met-GS scores in HCC patients from GEO dataset GSE15654 having good, intermediate or poor prognosis. **(E,F)** Dot-plots showing Alco_Met-GS scores in HCC patients from TCGA database representing different tumor stages (stage I, II or III) **(E)** and different tumor grades (G1, G2, or G3) **(F)**. **(G)** Dot-plot showing Cat-GS scores in HCC patients from TCGA database who experienced or did not experience vascular invasion. **(H,I)** Kaplan-Meier survival plots representing the percentage overall survival in HCC patients from GEO dataset GSE15654 (*n* = 216) **(H)** and from TCGA database (*n* = 370) **(I)** based on low vs. high Alco_Met-GS score. **(J)** Kaplan-Meier survival plot representing the percentage relapse free survival in HCC patients from TCGA database (*n* = 319) based on low vs. high Alco_Met-GS score. *P < 0.05; **P < 0.01; ns, not significant, Student's *t*-test.

To validate the significance of alcohol metabolism in HCC onset and progression, in general, and of ADH1A and ALDH2, in particular, we developed alcohol metabolism gene signature (Alco_Met-GS) comprised of ADH1A and ALDH2 (For details, see Materials and Methods section). Next, we verified that Alco_Met-GS is significantly downregulated in HCC tumors compared to adjacent normal tissues in patients from 14 different GEO datasets (Figure 5A). Recently, we and others have shown metabolic imbalance and hyperactive cell cycle signaling as key features of HCC onset and progression (Figure 1A and Figure S6A) (8, 59, 60); that's why we tested that whether Alco_Met-GS can predict overall molecular features of HCC. In this line, we found that gene sets associated with metabolic processes are enriched in HCC patients having a low Alco_Met-GS score compared to those having high Alco_Met-GS score from TCGA database and from different GEO datasets (Figure 5B). On the other hand, we found that gene sets associated with cell cycle progression were enriched in patients having low Alco_Met-GS scores as compared to those having high Alco_Met-GS scores (Figure S6B). Lastly, genes downregulated during liver cancer onset and progression are significantly enriched in patients having a high Alco_Met-GS score whereas the genes which are upregulated during liver cancer onset and progression are significantly enriched in patients having low Alco_Met-GS score (Figure 5C). All these results suggest that the suppression of alcohol metabolism associated Alco_Met-GS aligns well with HCC-specific molecular profile and can efficiently predict disease onset and progression in HCC.

### Alco_Met-GS Is Associated With Less Aggressive Disease State and Good Survival in HCC Patients

To validate the clinical significance of alcohol metabolism in HCC onset and progression, in general, and of ADH1A and ALDH2, in particular, we checked whether Alco_Met-GS is associated with clinical characteristics of HCC. In this line, we found that Alco_Met-GS is negatively correlated with prognosis in HCC as Alco_Met-GS score is low in patients having intermediate or poor prognosis compared to those having good prognosis (Figure 5D). We also observed low Alco_Met-GS score in Stage III and Grade 3 tumors compared to Stage I and Grade 1 tumors, respectively (Figures 5E,F). In addition, we observed significantly low Alco_Met-GS score in patients who experienced vascular invasion compared to those having no signs of vascular invasion (Figure 5G). Notably, we found that high Alco_Met-GS score is associated with good overall survival and relapse free survival in HCC patients from GEO dataset GSE15654 and TCGA database (Figures 5H–J).

## Discussion

In this study, we identified metabolic pathways associated with amino acid, lipid, carbohydrate, drug, and retinol metabolism as deregulated in HCC tumors compared to normal adjacent tissues by analyzing the gene expression data of HCC patients from different GEO datasets (Figure 1). Interestingly, we identified that catabolic enzymes coding genes associated with abovementioned pathways were mainly downregulated in HCC (Figure 1 and Figure S1). Amino acids serve as the building blocks for protein synthesis. Decreased catabolic degradation of amino acids leads to an excessive amino acid pool; thereby, it helps in accomplishing the protein production requirements of actively dividing cancer cells (61). Liver is the metabolic hub of the body which plays a vital role in lipid and fatty acid metabolism. Aberrantly activated lipogenesis due to deregulated lipid metabolism has been proposed as a key factor in regulating tumor growth and metastasis in HCC (62–64). Although proliferating cells acquire accelerated glycolysis profile to meet high energy demands along the path of tumorigenesis, both decreased and increased carbohydrate metabolism have been implicated in HCC onset and progression (65, 66). Efficacy of anti-tumor agents to treat cancer is directly dependent on how efficiently the drug is metabolized in the body. Low expression and activity of drug metabolizing enzymes have been observed in HCC leading to decreased tumor response and increased toxicity (67, 68). Similarly, aberrant metabolism of retinol or vitamin A, in general, has been identified as key process involved in hepatic fibrosis leading to HCC onset and progression (69).

Given the diverse roles played by mTOR signaling in cell growth and metabolism, its importance in HCC onset and progression cannot be overlooked. Notably, multiple upstream regulators of mTOR (IGF-1R, RAF, PI3K, PDK1, p-Akt), its binding partners (Rictor, Raptor) and immediate downstream effectors (4EBP1, S6K1, S6) have been reported to be dysregulated in HCC [(70); Figure 3C]. Despite their good performance in limiting tumor growth in preclinical and clinical studies, dose limiting toxicities such as diarrhea, skin rash, and an increase of transaminase have been found to be associated with pharmacological inhibitors of mTOR signaling (44). In addition, blocking mTORC1 activity inevitably results in therapy resistance mainly due to balance-shift toward mTORC2 activity which, in turn, boosts mTORC1 signaling via directly activating Akt at the upstream of mTORC1 (71). That's why a better understanding of signal transduction and identification of distant molecular targets at the downstream of mTOR signaling pathway is necessary to device novel therapeutic approaches to treat HCC.

**Figure 6:**
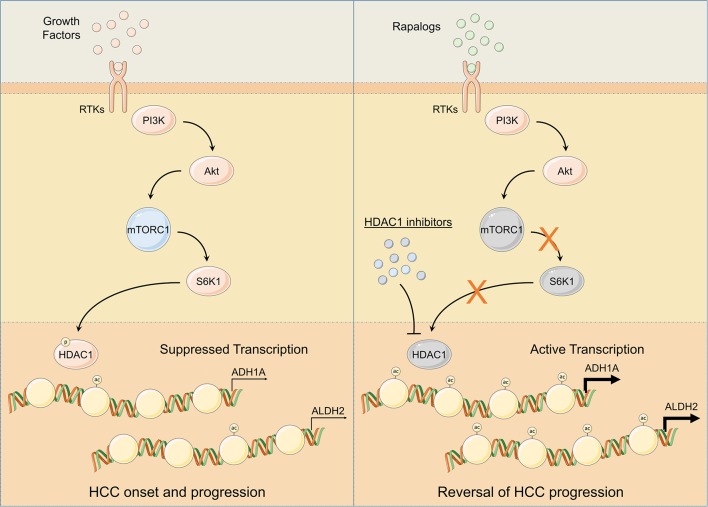
Schematic representation of mTOR signaling mediated regulation of ADH1A and ALDH2 expression. Following injury or damage, hepatocytes upregulate growth factor production. Upon RTK activation by growth factors, PI3K/Akt/mTOR signaling gets activated. Subsequent activation of S6K1 controls HDAC1 phosphorylation and promotes ADH1A and ALDH2 promoter deacetylation; thus, suppressing ADH1A and ALDH2 gene expression leading to HCC onset and progression **(Left)**. Pharmacological inhibition of HDAC1 using HDAC1 inhibitors and/or of mTOR signaling using rapalogs promotes ADH1A and ALDH2 gene expression resulting in reversal of HCC progression **(Right)**.

Our *in silico* findings suggest alcohol metabolism associated genes, ADH1A and ALDH2, as being transcriptionally suppressed by HDAC1 at the downstream of mTORC1 signaling leading toward HCC onset and progression (Figures 4, 6). HDAC1 has been established as directly regulated by mTORC1 signaling through mTOR-S6K1 axis (54) and we also found a positive correlation between mTOR signaling score and HDAC1 activity score (Figure 4L and Figure S5A) and between protein expression of S6K1 and HDAC1 activity score (Figure S5B) in HCC tumors. A significantly higher expression of HDACs have already been previously observed in HCC tumors compared to normal tissues, and their pharmacological inhibition resulted in inhibition of metastasis and invasion in HCC via upregulation of early growth response gene-1 and claudin-3 (72). We also found HDAC1 activity associated with aggressive disease states and poor survival in HCC patients (Figures S5C–G). As this study is limiting in terms of *in vitro* and *in vivo* validation, we could only show non-specific pharmacological inhibition of HDAC1 and its effects on the expression of downstream targets, ALDH1A and ALDH2 (Figures 4N–U), but we highly suggest that, in future, these findings should be validated *in vitro* and *in vivo* using HDAC1 specific inhibitors as well as with HDAC1 knockdown/knockout experiments.

Deregulated alcohol metabolism due to inhibition of ADH1A and ALDH2 at the downstream of mTORC1 signaling (Figure 4) can lead to an accumulation of acetaldehyde, an intermediate product of alcohol metabolism. Acetaldehyde, when not metabolized, is known to form DNA and protein adducts; thereby, (1) threatening DNA integrity by incorporating replication errors, (2) impairing DNA repair mechanisms, and (3) affecting other metabolic pathways by damaging mitochondria (48), cumulatively leading toward HCC onset. Consistent downregulation of Alco_Met-GS in HCC tumors compared to adjacent normal tissues in patients from multiple datasets (Figure 5A), its ability to accurately distinguish between key features of HCC onset and progression (Figure 5B and Figure S6B), and its association with less aggressive disease state and good overall survival (Figures 5C–J) strongly suggest decreased expression of ADH1A and ALDH2 to be used as biomarker of disease onset and progression in HCC. Overall, our *in silico* findings suggest that transcriptional suppression of alcohol metabolism regulators, ADH1A and ALDH2, at the downstream of mTOR signaling is, in part, responsible for triggering oncogenic transformation of hepatocytes resulting in disease onset and progression in HCC.

## Data Availability Statement

Publicly available datasets were analyzed in this study. This data can be found here: NCBI GEO datasets: GSE12941, GSE15654, GSE17856, GSE20017, GSE25097, GSE29721, GSE36376, GSE37129, GSE39791, GSE43619, GSE45436, GSE47197, GSE54236, GSE55092, GSE57555, GSE57957, GSE59713, GSE62232, GSE64041, GSE76297, GSE76427, GSE77322, GSE79246, GSE84402, GSE84598, GSE85257, GSE87630, GSE37988, GSE44909, GSE57956, and HCC patients' data from TCGA database.

## Author Contributions

KZ did KEGG pathway enrichment and clinical data analyses and drafted the manuscript. SY did correlation analyses, GSEAs and revised the manuscript. AK helped in downloading the data, and in calculating patient and pathway scores. UR downloaded the data, did differential expression, pathway enrichment and survival analyses, constructed the network, conceived the study, participated in its design and coordination, and drafted the manuscript. DG conceived the study, participated in its design and coordination, and critically reviewed the manuscript. Al authors read and approved the final manuscript.

### Conflict of Interest

The authors declare that the research was conducted in the absence of any commercial or financial relationships that could be construed as a potential conflict of interest.
