# Supplementary material for: mTOR/HDAC1 Crosstalk Mediated Suppression of ADH1A and ALDH2 Links Alcohol Metabolism to Hepatocellular Carcinoma Onset and Progression in silico
Source: Front Oncol. 2019 Oct 4;9:1000. doi: 10.3389/fonc.2019.01000 (PMC6787164; doi:10.3389/fonc.2019.01000)
Supplement: Supplementary file 1 [file Data_Sheet_1.pdf]

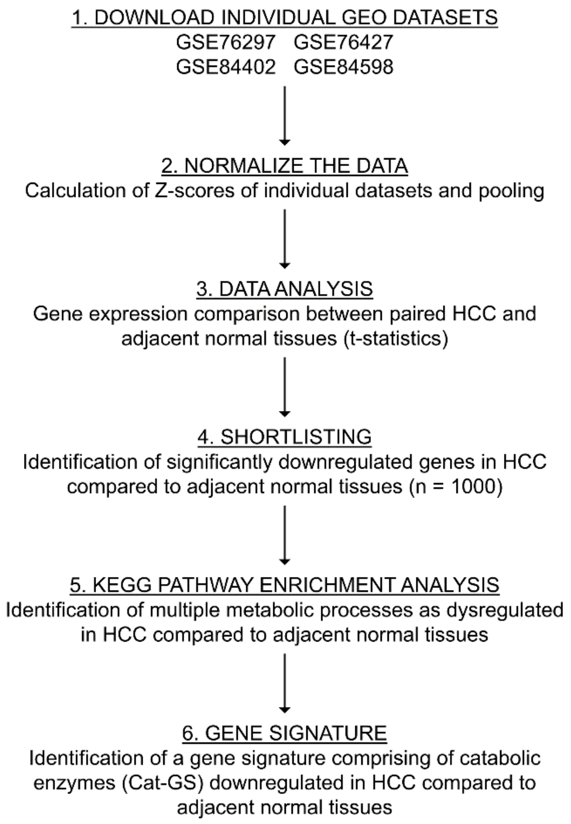

Fig. S1 Flowchart of identifying catabolic gene signature.

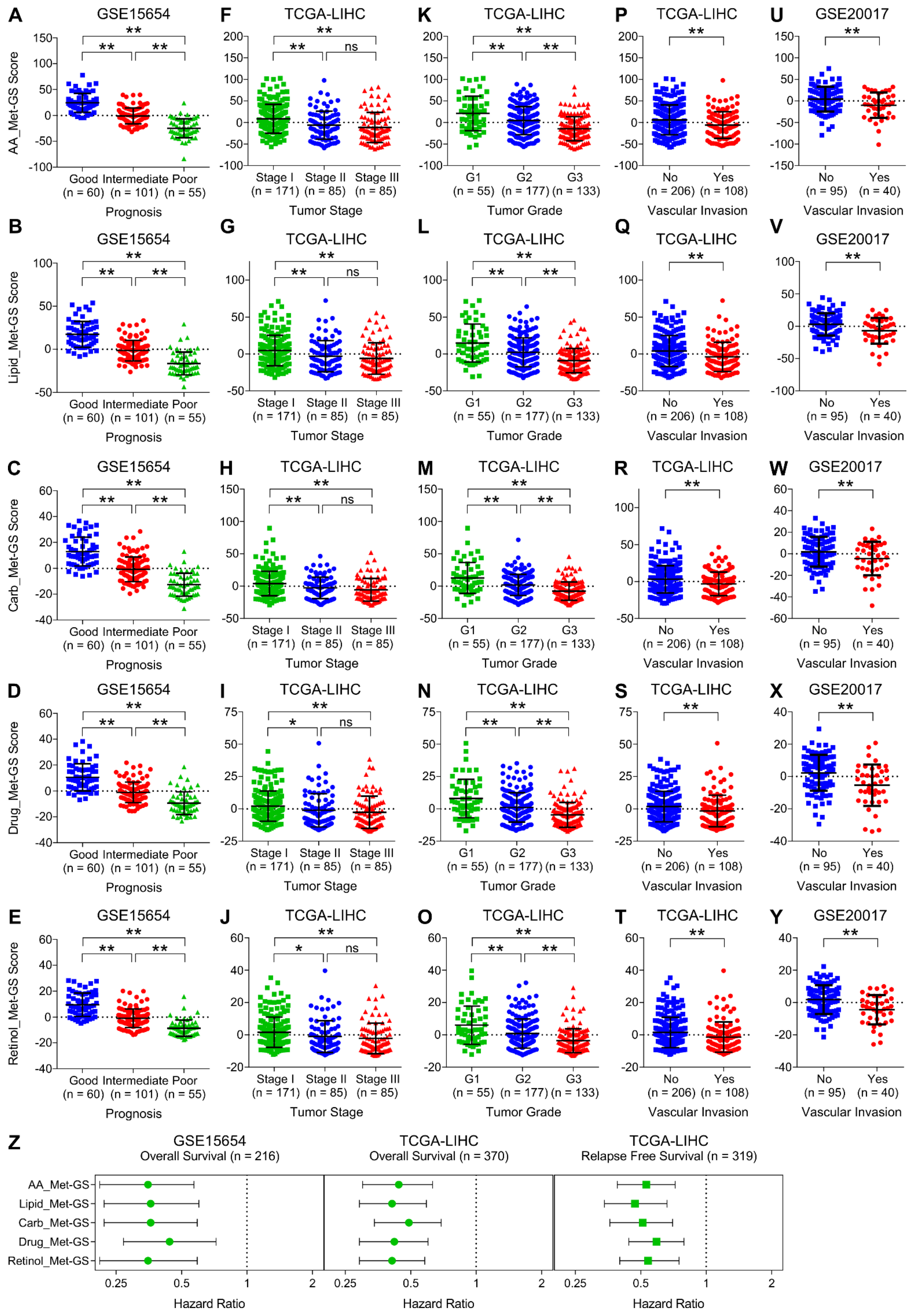
A GSE15654
AA_Met-GS Score
Good Intermediate Poor
(n = 60) (n = 101) (n = 55)
Prognosis
F TCGA-LIHC
Stage I Stage II Stage III
(n = 171) (n = 85) (n = 85)
Tumor Stage
K TCGA-LIHC
G1 G2 G3
(n = 55) (n = 177) (n = 133)
Tumor Grade
P TCGA-LIHC
No Yes
(n = 206) (n = 108)
Vascular Invasion
U GSE20017
No Yes
(n = 95) (n = 40)
Vascular Invasion
B GSE15654
Lipid_Met-GS Score
G TCGA-LIHC
L TCGA-LIHC
Q TCGA-LIHC
V GSE20017
C GSE15654
Carb_Met-GS Score
H TCGA-LIHC
M TCGA-LIHC
R TCGA-LIHC
W GSE20017
D GSE15654
Drug_Met-GS Score
I TCGA-LIHC
N TCGA-LIHC
S TCGA-LIHC
X GSE20017
E GSE15654
Retinol_Met-GS Score
J TCGA-LIHC
O TCGA-LIHC
T TCGA-LIHC
Y GSE20017
**
*
ns
Z
GSE15654
Overall Survival (n = 216)
TCGA-LIHC
Overall Survival (n = 370)
TCGA-LIHC
Relapse Free Survival (n = 319)
AA_Met-GS
Lipid_Met-GS
Carb_Met-GS
Drug_Met-GS
Retinol_Met-GS
0.25 0.5 1 2
Hazard Ratio

Fig. S2 AA_Met-GS, Lipid_Met-GS, Carb_Met-GS, Drug_Met-GS and Retinol_Met-GS are assoaciated with less aggressive disease state and good survival in HCC. (A-E) Dot-plots showing AA_Met-GS (A), Lipid_Met-GS (B), Carb_Met-GS (C), Drug_Met-GS (D) and Retinol_Met-GS (E) scores in HCC patients from GEO dataset 15654 having good, intermediate or poor prognosis. (F-J) Dot-plots showing AA_Met-GS (F), Lipid_Met-GS (G), Carb_Met-GS (H), Drug_Met-GS (I) and Retinol_Met-GS (J) scores in HCC patients from TCGA database representing different tumor stages (stage I, II or III). (K-O) Dot-plots showing AA_Met-GS (K), Lipid_Met-GS (L), Carb_Met-GS (M), Drug_Met-GS (N) and Retinol_Met-GS (O) scores in HCC patients from TCGA database representing different tumor grades (G1, G2 or G3). (P-T) Dot-plots showing AA_Met-GS (P), Lipid_Met-GS (Q), Carb_Met-GS (R), Drug_Met-GS (S) and Retinol_Met-GS (T) scores in HCC patients from TCGA database who experienced or did not experience vascular invasion. (U-Y) Dot-plots showing AA_Met-GS (U), Lipid_Met-GS (V), Carb_Met-GS (W), Drug_Met-GS (X) and Retinol_Met-GS (Y) scores in HCC patients from GEO dataset GSE20017 who experienced or did not experience vascular invasion. (Z) Forrest plots showing percentage overall survival (n=216) in HCC patients from GEO dataset GSE15654, and percentage overall survival (n=370) and percentage relapse free survival (n=319) in HCC patients from TCGA database based on AA_Met-GS, Lipid_Met-GS, Carb_Met-GS, Drug_Met-GS and Retinol_Met-GS score. *P <0.05; **P < 0.01; ns, not significant, Student's t-test.

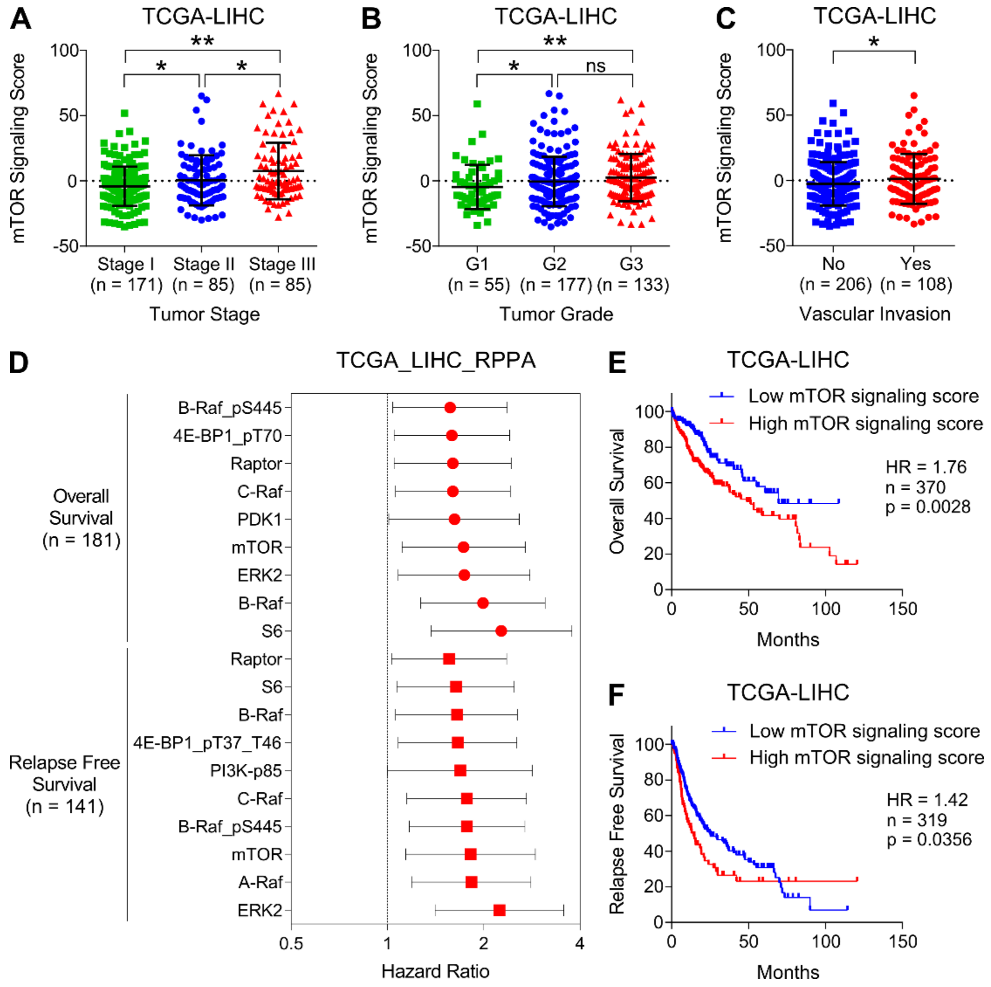

Fig. S3 mTOR signaling is associated with aggressive disease state and poor survival in HCC. (A and B) Dot-plots showing mTOR signaling score in HCC patients from TCGA database representing different tumor stages (stage I, II or III) (A) and different tumor grades (G1, G2 or G3) (B). (C) Dot-plot showing mTOR signaling score in HCC patients from TCGA database who experienced or did not experience vascular invasion. (D) Forrest plot showing percentage overall survival (n=181) and percentage relapse free survival (n=141) in HCC patients from TCGA database based on protein expression of genes associated with mTOR signaling. (E and F) Kaplan-Meier survival plots representing the percentage overall survival (n=370) (E) and percentage relapse free survival (n=319) (F) in HCC patients from TCGA database based on low vs high mTOR signaling score. *P <0.05; **P < 0.01; ns, not significant, Student's t-test.

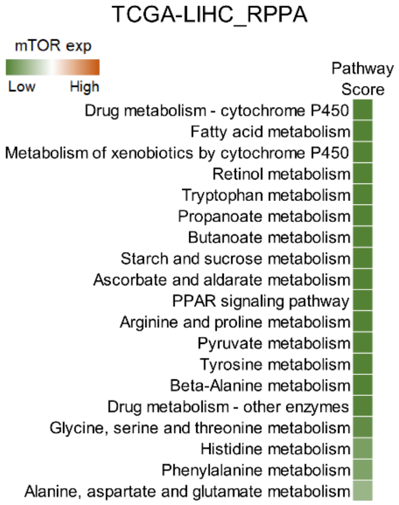

Fig. S4 Metabolic pathways are suppressed in patients with high mTOR expression. Heatmap showing enrichment of metabolic pathways associated gene sets in patients from TCGA database having low or high mTOR protein expression.

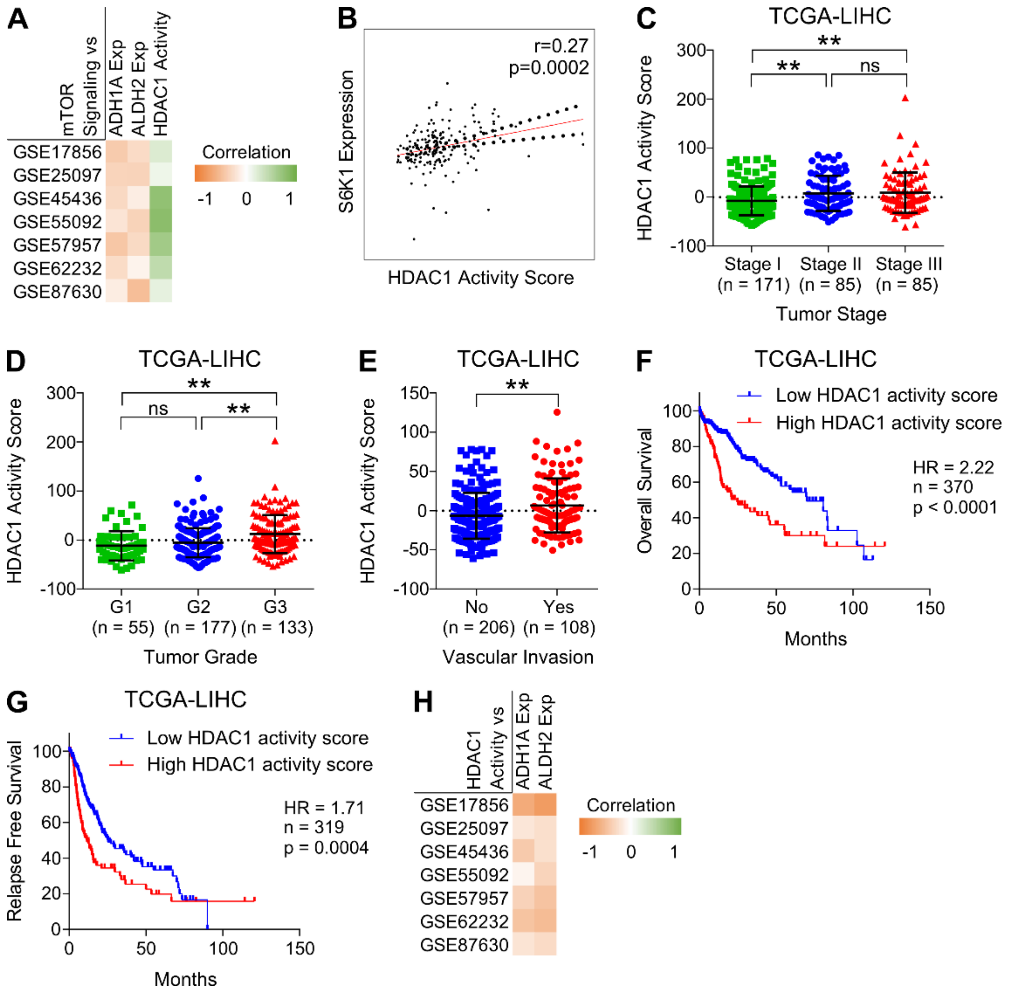

Fig. S5 HDAC1 activity is inversely correlated with ADH1A and ALDH2 expression, and is associated with aggressive disease state and poor survival in HCC at the downstream of mTOR signaling. (A) Heatmaps showing correlation of mTOR signaling score with ADH1A and ALDH2 expression, and HDAC1 activity score in patients from 6 different GEO datasets. (B) Graph showing correlation of S6K1 protein expression with with HDAC1 activity score in patients from TCGA database. (C and D) Dot-plots showing HDAC1 activation score in HCC patients from TCGA database representing different tumor stages (stage I, II or III) (C) and different tumor grades (G1, G2 or G3) (D). (E) Dot-plot showing HDAC1 activation score in HCC patients from TCGA database who experienced or did not experience vascular invasion. (F and G) Kaplan-Meier survival plots representing the percentage overall survival (n=370) (F) and percentage relapse free survival (n=319) (G) in HCC patients from TCGA database based on low vs high HDAC1 activation score. (H) Heatmap showing correlation of HDAC1 activity score with ADH1A and ALDH2 expression in patients from 6 different GEO datasets. *P <0.05; **P < 0.01; ns, not significant, Student’s t-test.

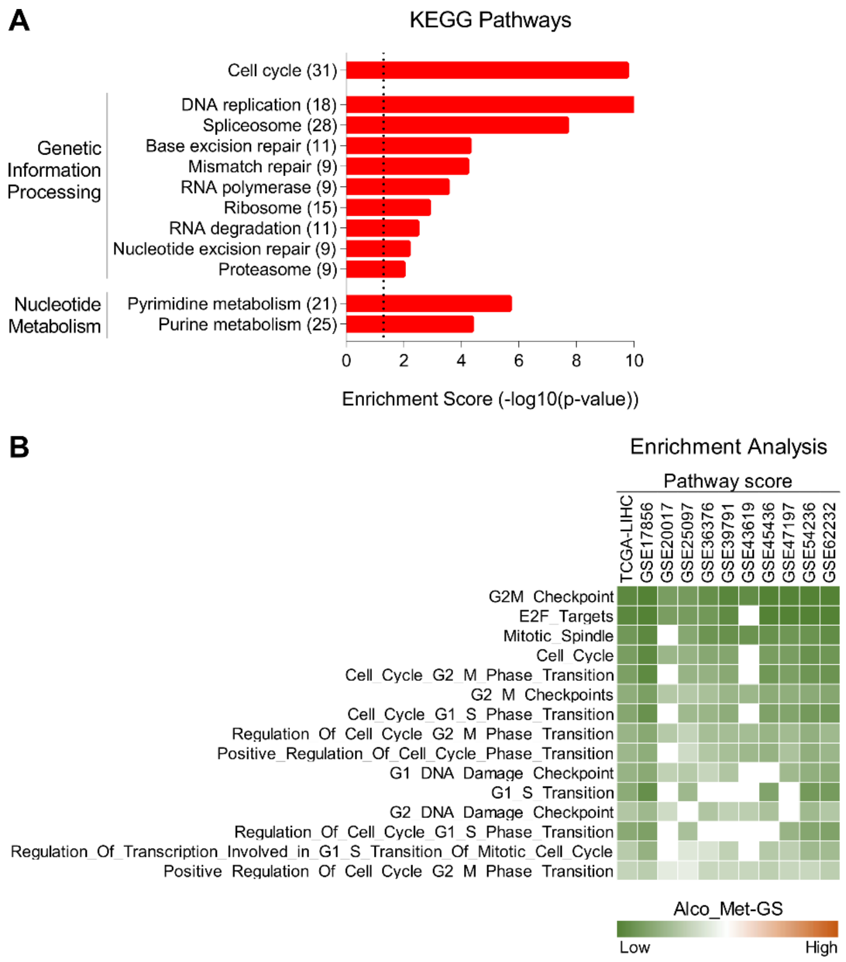

Fig. S6 Cell cycle progression is suppressed in patients having high Alco_Met-GS score. (A) Bar-graph showing top KEGG pathways associated with genes upregulated in HCC compared to adjacent normal tissues. (B) Heatmap showing enrichment of cell cycle progression associated gene sets in patients from TCGA database and from 10 different GEO datasets having low or high Alco_Met-GS.

Table S1 Summary of patient's datasets analyzed for molecular and clinico-pathological comparisons. List of patients' datasets is provided along with the information about number of samples available of each tissue type (Normal and/or Tumor) and type of profiling data (methylation, gene expression and/or RPPA) and clinico-pathological data (prognosis, stage, grade, vascular invasion and/or survival) analyzed from each dataset. +: Yes, -: No.

	Tissue Type (N)		Profiling data analyzed			Clinico-pathological data analyzed				
**Dataset**	**Normal**	**Tumor**	**Methylation**	**Gene expression**	**RPPA**	**Prognosis**	**Stage**	**Grade**	**Vascular invasion**	**Survival**
**TCGA**	0	371	-	+	+	-	+	+	+	+
**GSE12941**	10	10	-	+	-	-	-	-	-	-
**GSE15654**	0	216	-	+	-	+	-	-	-	+
**GSE17856**	40	40	-	+	-	-	-	-	-	-
**GSE20017**	0	135	-	+	-	-	-	-	+	-
**GSE25097**	243	268	-	+	-	-	-	-	-	-
**GSE29721**	10	10	-	+	-	-	-	-	-	-
**GSE36376**	193	240	-	+	-	-	-	-	-	-
**GSE37988**	62	62	+	-	-	-	-	-	-	-
**GSE39791**	72	72	-	+	-	-	-	-	-	-
**GSE44909**	12	12	+	-	-	-	-	-	-	-
**GSE45436**	41	93	-	+	-	-	-	-	-	-
**GSE47197**	61	61	-	+	-	-	-	-	-	-
**GSE55092**	81	39	-	+	-	-	-	-	-	-
**GSE57555**	16	16	-	+	-	-	-	-	-	-
**GSE57956**	57	57	+	-	-	-	-	-	-	-
**GSE57957**	37	37	-	+	-	-	-	-	-	-
**GSE62232**	10	81	-	+	-	-	-	-	-	-
**GSE64041**	60	60	-	+	-	-	-	-	-	-
**GSE76297**	59	59	-	+	-	-	-	-	-	-
**GSE76427**	52	52	-	+	-	-	-	-	-	-
**GSE84402**	14	14	-	+	-	-	-	-	-	-
**GSE84598**	22	22	-	+	-	-	-	-	-	-
**GSE87630**	30	64	-	+	-	-	-	-	-	-

Table S2 Survival analyses of genes in Cat-GS. Survival analysis results of individual genes in Cat-GS using data of HCC patients from TCGA database. HR: Hazard Ratio, N; Number of patients.

Gene Symbol	HR	p-value	N
AADAT	0.76	0.12304	370
ACAA1	0.75	0.11440	370
ACACB	0.78	0.16458	370
ACADL	0.70	0.04885	370
ACADS	0.61	0.00670	370
ACADVL	0.74	0.08690	370
ACAT1	0.61	0.00545	370
ACOT12	0.59	0.00271	370
ACOX2	0.71	0.05438	370
ACSM2A	0.66	0.01870	370
ACSM3	0.61	0.00569	370
ACSM5	0.64	0.01340	370
ACY1	0.70	0.04881	370
ADH1A	0.60	0.00501	370
ADH1B	0.73	0.08086	370
ADH1C	0.63	0.00969	370
ADH4	0.52	0.00026	370
ADH6	0.70	0.04254	370
AKR1D1	0.59	0.00324	370
ALDH1B1	0.88	0.46572	370
ALDH2	0.64	0.01164	370
ALDH4A1	0.74	0.09276	370
ALDH9A1	0.66	0.02084	370
AMDHD1	0.60	0.00457	370
AMT	0.90	0.53860	370
AOC3	0.76	0.12015	370
ARG1	0.79	0.18608	370
ASPA	0.53	0.00048	370
BBOX1	0.76	0.12772	370
BDH2	0.76	0.11753	370
CAT	0.54	0.00068	370
CDA	1.04	0.81570	370
COMT	0.84	0.31781	370
CPT2	0.76	0.11606	370
CYP1A2	0.91	0.60675	370
CYP26A1	0.98	0.90787	370
CYP27A1	0.62	0.00759	370
CYP2A7	0.87	0.42188	370
CYP2C19	0.54	0.00056	370
CYP2C8	0.55	0.00080	370
CYP2C9	0.54	0.00058	370
CYP2E1	0.66	0.02226	370
CYP2J2	0.79	0.18174	370
CYP39A1	0.91	0.58466	370
CYP3A43	0.54	0.00054	370
CYP3A5	0.57	0.00143	370
CYP4A11	0.68	0.03168	370
CYP4F2	0.65	0.01404	370
CYP4F3	0.69	0.03717	370
CYP8B1	0.62	0.00846	370
DAO	0.67	0.02318	370
DBT	0.59	0.00335	370
DMGDH	0.45	0.00001	370
DPYS	0.60	0.00447	370
ECHS1	0.65	0.01544	370
ENO3	0.80	0.22180	370

EPHX2	0.66	0.01935	370
FAH	0.76	0.12329	370
FBP1	0.67	0.02252	370
FMO2	0.68	0.03334	370
FMO3	0.60	0.00387	370
FTCD	0.54	0.00055	370
GAMT	0.62	0.00831	370
GCAT	0.85	0.35691	370
GCDH	0.60	0.00502	370
GGT5	0.65	0.01440	370
GLYCTK	0.64	0.01112	370
GNMT	0.61	0.00484	370
GOT1	0.86	0.39726	370
GOT2	0.49	0.00006	370
GPT	0.57	0.00149	370
GPT2	0.62	0.00732	370
GSTZ1	0.81	0.22579	370
HAAO	0.67	0.02516	370
HADH	0.68	0.03173	370
HAGH	0.60	0.00413	370
HGD	0.62	0.00762	370
HK3	1.19	0.33363	370
HMGCL	0.72	0.06172	370
HMGCS2	0.47	0.00003	370
HPD	0.70	0.04358	370
IDO2	0.82	0.26451	370
INMT	0.70	0.04360	370
IVD	0.55	0.00083	370
KMO	0.77	0.13875	370
LDHD	0.66	0.01829	370
MAT1A	0.73	0.08337	370
MLYCD	0.74	0.09316	370
MUT	0.55	0.00086	370
NAT2	0.69	0.04047	370
OGDHL	0.66	0.01784	370
PC	0.99	0.94914	370
PGM1	0.62	0.00822	370
PIPOX	0.63	0.01058	370
PLA2G5	0.77	0.13362	370
PNPLA4	0.75	0.10558	370
PRODH2	0.79	0.19117	370
RDH16	0.59	0.00295	370
RETSAT	0.73	0.07565	370
SARDH	0.54	0.00071	370
SAT2	0.67	0.02296	370
SDS	0.63	0.00903	370
SUCLG2	0.67	0.02553	370
TAT	0.52	0.00025	370
TDO2	0.83	0.29370	370
TPMT	0.72	0.06287	370
UPB1	0.51	0.00017	370
UPP2	0.73	0.08111	370
UROC1	0.63	0.00957	370
XDH	0.62	0.00674	370
